# Metataxonomic and Metabolic Impact of Fecal Microbiota Transplantation From Patients With Pancreatic Cancer Into Germ-Free Mice: A Pilot Study

**DOI:** 10.3389/fcimb.2021.752889

**Published:** 2021-10-19

**Authors:** Laurence Genton, Vladimir Lazarevic, Ozren Stojanovic, Martina Spiljar, Souad Djaafar, Thibaud Koessler, Valérie Dutoit, Nadia Gaïa, Julie Mareschal, Andrew James Macpherson, Francois Herrmann, Mirko Trajkovski, Jacques Schrenzel

**Affiliations:** ^1^ Clinical Nutrition, University Hospitals of Geneva and University of Geneva, Geneva, Switzerland; ^2^ Genomic Research Laboratory, University of Geneva, Geneva, Switzerland; ^3^ Department of Cell Physiology and Metabolism, University of Geneva, Geneva, Switzerland; ^4^ Oncology, University Hospitals of Geneva and University of Geneva, Geneva, Switzerland; ^5^ Laboratory of Tumor Immunology and Translational Research Center for Oncoheamatology, University of Geneva, Geneva, Switzerland; ^6^ Department of Biomedical Research, University Hospital of Bern and University of Bern, Bern, Switzerland; ^7^ Rehabilitation and Geriatrics, University Hospitals of Geneva and University of Geneva, Geneva, Switzerland; ^8^ Infectious Diseases, University Hospitals of Geneva and University of Geneva, Geneva, Switzerland

**Keywords:** microbiota, visceral fat, muscle, weight, fecal material transplantation, pancreatic cancer, mice

## Abstract

**Background:**

Body weight (BW) loss is prevalent in patients with pancreatic cancer (PC). Gut microbiota affects BW and is known to directly shape the host immune responses and antitumor immunity. This pilot study evaluated the link between gut microbiota, metabolic parameters and inflammatory/immune parameters, through the fecal material transplantation (FMT) of PC patients and healthy volunteers into germ-free (GF) mice.

**Methods:**

We transplanted the feces from five PC patients and five age- and gender-matched healthy volunteers into two GF mice each. Mouse BW and energy intake were measured every 1-5 days, oral glucose on day 21, insulin tolerance on day 26, fecal bacterial taxonomic profile by 16S rRNA gene sequencing on day 5, 10, 15 and 30, and gut-associated lymphoid tissue T cells, plasma cytokines and weights of fat and muscle mass at sacrifice (day 34). Results are presented as mean ± SD. The continuous parameters of mice groups were compared by linear univariate regressions, and their bacterial communities by Principal Coordinates Analysis (PCoA), Bray-Curtis similarity and ANCOM test.

**Results:**

Recipients of feces from PC patients and healthy volunteers had similar BW gain and food intake. Visceral fat was lower in recipients of feces from PC patients than from healthy individuals (0.72 ± 0.17 *vs.* 0.92 ± 0.14 g; coeff -0.19, 95% CI -0.38, -0.02, p=0.035). The other non-metataxonomic parameters did not differ between groups. In PCoA, microbiota from PC patients clustered apart from those of healthy volunteers and the same pattern was observed in transplanted mice. The proportions of *Clostridium bolteae*, *Clostridium scindens*, *Clostridium*_g24_unclassified and *Phascolarctobacterium faecium* were higher, while those of *Alistipes obesi*, Lachnospiraceae PAC000196_s and Coriobacteriaceae_unclassified species were lower in PC patients and in mice transplanted with the feces from these patients.

**Conclusion:**

In this pilot study, FMT from PC patients was associated with a decrease in visceral fat as compared to FMT from healthy individuals. Some of the differences in fecal microbiota between PC and control samples are common to humans and mice. Further research is required to confirm that feces contain elements involved in metabolic and immune alterations.

## Introduction

Pancreatic cancer (PC) is the 7^th^ cause of death by cancer worldwide (Arnold et al., 2020), the 3^rd^ in the US in the 2021 estimate (https://cancerstatisticscenter.cancer.org/#!/) and is foreseen to be the second cause of cancer death by 2030 (Rahib et al., 2014). It is often detected late, at a metastatic or locally advanced stage, and is associated with a 5-year survival of around 10%. The most commonly identified risk factors are smoking, chronic pancreatitis, type 2 diabetes, obesity and age.

Human studies suggest that microorganisms colonize the pancreas in pancreatic diseases and in the healthy state (Thomas and Jobin, 2020). This could occur *via* the pancreatic duct or the portal circulation after translocation from the digestive tract through the gut barrier (Thomas and Jobin, 2020). Indeed, the gut microbiome reflects 25% of the tumor microbiome (Riquelme et al., 2019). A few cross-sectional studies described differences in abundance and composition of fecal or rectal bacteria when comparing PC patients with healthy controls, although no consensus could be established between the studies (Ren et al., 2017; Pushalkar et al., 2018; Half et al., 2019).

Animal studies have shown involvement of gut microbiota in tumor progression. Germ-free (GF) mice or mice treated with antibiotics are less prone to tumor growth and oncogenesis (Schwabe and Jobin, 2013; Thomas et al., 2018). In murine models of PC, fecal Actinobacteria, Deferribacteres and *Bifidobacterium* increase in abundance with time, and bacterial ablation through oral antibiotics limited the tumor growth (Pushalkar et al., 2018). It was suggested that the gut microbiota triggers an inflammatory response promoting pancreatic cancer initiation (Schwabe and Jobin, 2013). Recently, a translational study evaluated the impact of fecal material transplantation (FMT) from surgically-resected PC patients into antibiotic-treated PC-bearing mice (Riquelme et al., 2019). Mice that received the stool of long-term survivors (> 5 years) showed a slower tumor growth, a higher survival, higher serum levels of interferon-γ and interleukin-2 than mice that had received the feces of short-term survivors, and displayed a distinct gut microbiota community. This anti-tumoral effect was mediated by CD8+ T cells. Taken together, these animal studies suggest that the modulation of gut microbiota, whether through FMT or antibiotics, affects the immune and inflammatory parameters and the pancreatic tumor growth. In addition, it has been shown that gut microbiota influences immune responses to cancer in patients, including response to immune checkpoint inhibitors (Routy et al., 2018; Gopalakrishnan et al., 2018). In pancreatic cancer, it was shown that tumors harbor more microbes than the normal pancreatic tissue (Sethi et al., 2019) and that the tumor microbiome promotes immunosuppression at the tumor microenvironment (Pushalkar et al., 2018). Additionally, the PC microbiome composition was shown to influence antitumor immune responses, allowing identification of tumor microbiome signatures predicting of PC patients' outcome (Riquelme et al., 2019).

Although obesity is a risk factor for developing pancreatic cancer, 60 to 70% of the patients suffer of cachexia at diagnosis of PC, defined as a loss of body weight and muscle mass. Cachexia is mediated by anorexia, insulin resistance, systemic inflammation and likely increased brown adipose tissue (Tsoli and Robertson, 2013), and is associated with a high morbidity and mortality (Muscaritoli et al., 2017; Hendifar et al., 2018). Gut microbiota has been associated with body weight and body composition in animal studies. Germ-free mice suffer from muscle atrophy, which improves after FMT from pathogen-free mice (Lahiri et al., 2019). Translational studies have shown that body composition phenotypes, whether obesity or leanness, are transmissible by FMT from humans to GF mice (Ridaura et al., 2013; Blanton et al., 2016). However, it is unknown whether a FMT from PC patients, prone to cachexia, induces changes in body composition of GF mice.

We hypothesized that the gut microbiota of patients with newly diagnosed PC, naïve of any oncologic treatment, induced alterations of metabolic, inflammatory and immune parameters. If these hypotheses were confirmed, they would pave the way for interventional human studies. In this pilot study, we evaluated whether patients with PC displayed a different microbiota composition compared to healthy controls, and whether FMT from these patients into GF mice affected body weight gain, body composition, as well as intestinal immune and inflammatory parameters.

## Study Population And Methods

This translational pilot study examined the microbiota alterations in patients with PC compared to healthy volunteers, and the impact of the FMT coming from these patients into GF mice. The human protocol was approved by the local Ethical Committee and the participants signed an informed consent. The animal experiments were approved by the Swiss Federal and the Geneva Cantonal Authorities for animal experimentation.

### Human Donors

The patients were recruited at the University Hospitals of Geneva. We included five patients aged ≥ 18 years, newly diagnosed with pancreatic adenocarcinoma, with no tube feeding nor parenteral nutrition, and without treatment with antibiotics in the previous month. We also included 5 healthy volunteers, matched for gender and age (+/- 5 years) with the included patients with PC, aged ≥ 18 years, with a body mass index below 30 kg/m^2^, no chronic diseases and no treatment with antibiotics in the previous month. The exclusion criteria for all donors were: age < 18 years, cognitive impairment, ongoing oncologic treatment, tube feeding or parenteral nutrition and antibiotic therapy.

For the patients with PC, we collected the following data at the time of inclusion: cancer stage (Kakar et al., 2016), date of diagnosis and results of routine blood samples (complete blood count, urea, creatinine, Na, K, aspartate aminotransferase, alanine aminotransferase, alkaline phosphatase, ɣ-glutamyl-transferase, albumin, C-reactive protein). For patients and volunteers, we reported drugs, co-morbidities and body weight loss over the last 6 months. We measured actual weight with an electronic scale (Seca, Hamburg, Germany) and height with a height gauge. Waist-to-hip ratio was determined with the patient or volunteer in a standing position, legs closed and arms alongside the body, at the level of the umbilicus of the waist and at the largest level of the hip. Body composition was assessed by 50 kHz tetrapolar bioelectrical impedance analysis (Nutriguard^®^, Data Input GmbH), with the patient or volunteer lying in a supine position, arms and legs abducted from the trunk at about 30 degrees. The measured resistance and reactance were used to calculate fat-free mass by the Geneva formula, validated against dual-energy X-ray absorptiometry in the healthy population living in the Geneva area (Kyle et al., 2001). Fat mass was obtained by subtraction of fat-free mass from body weight. Appetite was evaluated by a visual analogue scale, ranging from 0 (no appetite) to 100 mm (very good appetite) (Blauwhoff-Buskermolen et al., 2016).

Stools were collected within 7 days of the routine consultation during which the clinical assessments were performed. They were collected in a Commode Specimen Container (Covidien, Medline, Arnhem, The Netherlands) placed over the toilet seat opening. The participant put at least a nut-sized section of fecal material immediately into Sarstedt Feces Tube 76x20mm, sealed it in an AnaeroPouch-Bag (Thermo Scientific) with an AnaeroPouch-Anaero gas generator (Thermo Scientific), and the study investigators transported it within 4 h to the laboratory for further processing. About 0.5 mL stool sample was added to 4.5 mL of PBS-CR (phosphate buffer saline containing 0.1% cysteine and 0.3% riboflavin) and homogenized by vortexing during 3 min. To settle large insoluble particles, the sample tubes were allowed to stand for 3 minutes. The supernatant (900 µL) was then mixed with an equal volume of PBS-CRGS (PBS-CR with 30% glycerol and 10% sucrose). The suspension was stored in cryovial 100 µL-aliquots at –80°C until transplantation.

### Animal Recipients

The animal study was performed at the Medical School of the University of Geneva. The fecal suspension of each of the five patients with PC and the five healthy volunteers was transplanted into two GF mice (males, C57BL/6J, 7–8 weeks old, obtained from the University of Bern) by oral gavage on days 0, 2 and 6 (200 µL each day). The two mice that received the feces from the same donor were housed in the same cage. All mice were fed with a standard chow diet (SAFE 150, Safediets, Augy, France) and kept in specific pathogen free (SPF) conditions. The initial colonization of the GF mice with the respective microbiota was done immediately upon arrival, and all transplanted mice were kept in sterilized cages and handled in sterilized hoods using sterile single-use sterile protective gear and coating. Food intake was measured every 3–5 days, by cage.

Body weight was measured every 1 to 5 days. The oral glucose tolerance test (oGTT) was determined on day 21, after a 6-hour fast, by oral gavage of a glucose bolus (2 g/kg body weight). The insulin tolerance test (ITT) was performed on day 26 after a 5-hour fast, with an intraperitoneal injection of 0.75 U/kg insulin (ref. I9278, Sigma-Aldrich). Feces were collected on days 5, 10, 15 and 30 after the first FMT and frozen at –80°C until analysis. At the end of the experiment (day 34), we measured the weight of the subcutaneous, visceral and brown adipose tissue and the skeletal muscle mass. On day 34, the following serum cytokines/chemokines were measured using the 23-plex mouse cytokine & chemokine assay (Bio-Rad) in plasma: interleukin (IL)-1β, IL-2, IL-3, IL-4, IL-5, IL-6, IL-9, IL-10, IL-12 (p40), IL-12(p70), IL-13, IL-17A, eotaxin, G-CSF, GM-CSF, interferon-γ, KC, MCP-1, MIP1α, MIP-1β, RANTES and tumor necrosis factor-α. Finally, numbers of myeloid cells, CD4^+^ and CD8^+^ lymphocytes and T regulatory (Treg) cells were measured in the Peyer’s patches, mesenteric lymph nodes and blood by flow cytometry after surface staining using CD45, CD3, CD4, CD8, CD11b, and intracellular staining using FoxP3 and RORγt using the manufacturer’s instructions (Biolegend). Lymphocytes in the Peyer’s patches and mesenteric lymph nodes were isolated as previously described (Genton et al., 2004).

### Metataxonomic Analysis of Fecal Samples

DNA was extracted from 20–30 mg of mouse stools or 100 μL of human stool suspension using ZymoBIOMICS DNA Miniprep Kit (Zymo Research). Purified DNA was quantified using the Qubit dsDNA BR Assay Kit (Thermo Fisher Scientific) and stored at –20°C.

The V3–4 region of the bacterial 16S rRNA genes was amplified using 1 ng of extracted DNA as described previously (Somm et al., 2021). The construction of the sequencing library using the MetaFast protocol, the Illumina MiSeq 2×300 sequencing with MiSeq Reagent Kit v3 and initial sequence processing (demultiplexing and removal of adapter and primer sequences) were carried out at Fasteris (Plan-les-Ouates, Switzerland). For the sequence analysis, paired reads were quality filtered and joined using PEAR v0.9.11 (-m 470, -n 390, -t 150, -v 10, -q 33, -p 0.0001, -u 0) (Zhang et al., 2014). Merged sequence reads were clustered into zero-radius operational taxonomic units (zOTUs) using UNOISE3 from the USEARCH v10.0.240 pipeline (Edgar, 2013; Edgar, 2016). zOTUs were classified using EzBioCloud 16S database (Yoon et al., 2017) *via* MOTHUR (Schloss et al., 2009) command classify.seqs (method=wang, cutoff=80). We removed the reads corresponding to zOTUs that had < 90% identity to the reference EzBioCloud 16S database as revealed by USEARCH (-id 0.90, -query_cov 0.99) (Edgar, 2010). Sequencing data were submitted to the European Nucleotide Archive (ENA; www.ebi.ac.uk/ena; study number: PRJEB43581).

### Statistics

Results are presented as mean ± SD. Normality of distributions was checked with Shapiro-Wilk tests. As plasma cytokines were not normally distributed, they were normalized using the Stata’s “ladder” command, in order to be used in the mixed regression models. Univariate linear regressions were performed to compare non-metataxonomic data between the animal recipients of feces from PC patients (10 mice) and volunteers (10 mice, reference group). They took into account that 2 mice were transplanted with the feces of the same human donor. For each regression model, we calculated the regression coefficients, standard errors and 95% confidence intervals (CI). Statistics were performed with STATA release 16.1, and significance was set at p<0.05.

Shannon diversity was calculated after rarefaction to 20’900 reads per sample using the rrarefy function of the R vegan package. Principal coordinates analysis (PCoA) of the Bray-Curtis similarity matrix were used to visualize microbiota similarities based on the square-root transformed zOTU and species abundances. Since each human feces was transplanted into two mice (denoted Group A and B), microbiota analyses were, when appropriate, performed twice. To compare the relative abundance of bacterial genera between human donors and corresponding mouse recipients, we used the analysis of composition of microbiomes (ANCOM) v2.1 (Mandal et al., 2015) with the following settings: adjusted = F, repeated = F, multcorr = 2 (“less stringent” multiple comparison correction), sig = 0.05, prev.cut = 0.75 (features not observed in ≥ 75% samples were omitted) and paired.test_ancom = paired. The results that passed the 0.6 threshold were considered significant. Wilcoxon rank sum test was used to compare Bray-Curtis similarities within and between groups defined with respect to the cancer status of human donors.

As this was a pilot study, no sample size calculation was performed. However, a sample size of 6 to 10 mice per group was used in a study which showed statistically significant differences in gut microbiota composition in mouse models of cancer (leukemia) as compared to control mice (Bindels et al., 2012).

## Results

**Figure 1:**
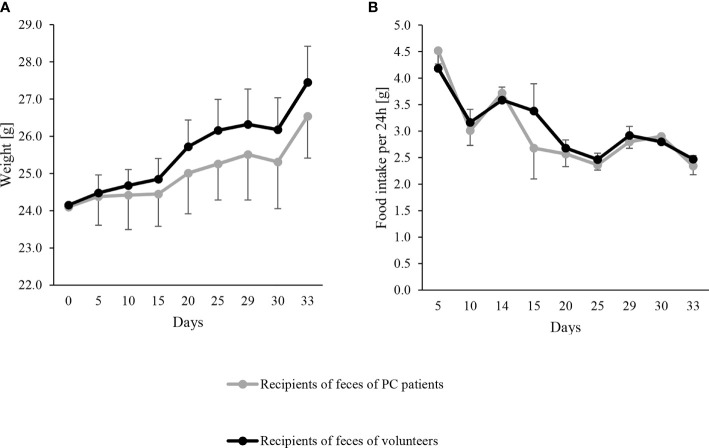
Evolution of body weight **(A)** and food intake **(B)** in the mouse recipients of feces from pancreatic cancer (PC) patients as mean – SD (gray line) (n = 10) and volunteers as mean + SD (black line) (n = 10). FMT were performed on day 0, 2 and 6. Body weight at each time point was never significantly different between both groups by linear regression models (all p > 0.5).

Characteristics of human donors are shown in **Table 1**. Mouse recipients of feces from PC patients tended to have a lower, albeit not significant, BW gain over the whole study than the recipients of healthy volunteer’s feces, (2.4 g *vs.* 3.3 g; coeff −0.86, 95% CI −1.83 to 0.83, p=0.069), while total food intake was similar between groups (49.1 g *vs.* 50.3 g, coeff −1.21, 95% CI −5.74 to 3.33, p=0.561). The evolution of BW and energy intake is shown in **Figure 1**. oGTT and ITT were similar in both groups (**Supplemental Figure 1**). At sacrifice, visceral fat was lower in recipients of feces from PC patients (**Table 2**) but the levels of subcutaneous and brown adipose tissue and skeletal muscle mass were similar between groups. There were no significant differences in plasma cytokines and chemokines, myeloid, T or Treg cells and Peyer’s patch numbers were similar between groups (**Supplemental Tables 1**, **2**).

**Table 1:** Baseline characteristics of PC patients and healthy volunteers.

	Patients with pancreatic cancer					Healthy volunteers				
Donor number	1	2	3	4	5	11	12	13	14	15
Gender [w/m]^a^	m	m	m	w	m	m	m	m	w	m
Age [years]	55	74	71	74	59	55	78	75	74	58
Staging of pancreatic cancer	III	IA	IV	IV	IV	–	–	–	–	–
**Anthropometry**										
Body mass index [kg/m^2^]	22.0	25.6	22.6	25.2	22.0	19.4	24.2	26.4	24.1	25.4
Fat-free mass index [kg/m^2^]^b^	17.8	18.3	17.2	13.9	16.6	17.0	18.7	16.5	18.2	18.3
Weight change over 6 months [kg]	-15.0	0.0	-0.6	-5.9	-6.2	0.0	2.5	3.0	0.3	5.0
Waist/hip ratio	0.92	1.10	0.98	0.89	0.97	0.77	0.96	0.93	1.03	0.96
Appetite [0-100]	35	70	45	30	60	86	90	90	70	64
**Plasma values**										
Hemoglobin [g/L]	85	144	147	119	149	–	–	–	–	–
Glucose [mmol/L]	–	7.2	6.0	–	–	–	–	–	–	–
Urea [mmol/L]	9.7	5.5	5.3	8.0	6.9	–	–	–	–	–
Creatinin [µmol/L]	132	90	91	61	82	–	–	–	–	–
Aspartate aminotransferase [U/L]	42	21	16	32	15	–	–	–	–	–
Alanine aminotransferase [U/L]	42	17	15	26	18	–	–	–	–	–
Alkaline phosphatase [U/L]	275	72	83	199	62	–	–	–	–	–
ɣ-glutamyl-transferase [U/L]	39	28	86	218	50	–	–	–	–	–
Albumin [g/L]	–	44	45	35	46	–	–	–	–	–
C-reactive protein [mg/L]	23.6	3.1	1.8	–	3.5	–	–	–	–	–

a w, woman; m, man.

b fat-free mass index = fat-free mass [kg]/height[m]^2^.

**Table 2:** Body composition of mouse recipients at sacrifice.

	Recipients of feces of PC patients			Recipients of feces of volunteers			Univariate linear regressions^b^		
	[n=10]			[n=10]					
	Mean	±	SD	Mean	±	SD	Coefficient	95% CI	p
Subcutaneous adipose tissue [g]	0.51	±	0.09	0.55	±	0.13	-0.04	[-0.16, 0.07]	0.416
Visceral adipose tissue [g]	0.72	±	0.17	0.92	±	0.14	-0.19	[-0.38, -0.02]	0.035
Brown adipose tissue [g]	0.12	±	0.03	0.13	±	0.02	-0.01	[-0.04, 0.01]	0.468
Skeletal muscle mass [g]^a^	0.64	±	0.03	0.64	±	0.06	-0.01	[-0.06, 0.05]	0.846

PC, pancreatic cancer; CI, 95% confidence interval.

a Skeletal muscle mass = muscle mass of both quadriceps, gastrocnemius and soleus.

b Univariate linear regressions comparing body composition between mouse recipients of feces of 5 PC patients vs. 5 healthy volunteers. They take into account that two mice were transplanted with the feces of the same human donor. The reference group are the mice that received the feces of the healthy volunteers.

### Microbiota of PC Patients *vs.* That of Volunteers

**Figure 2:**
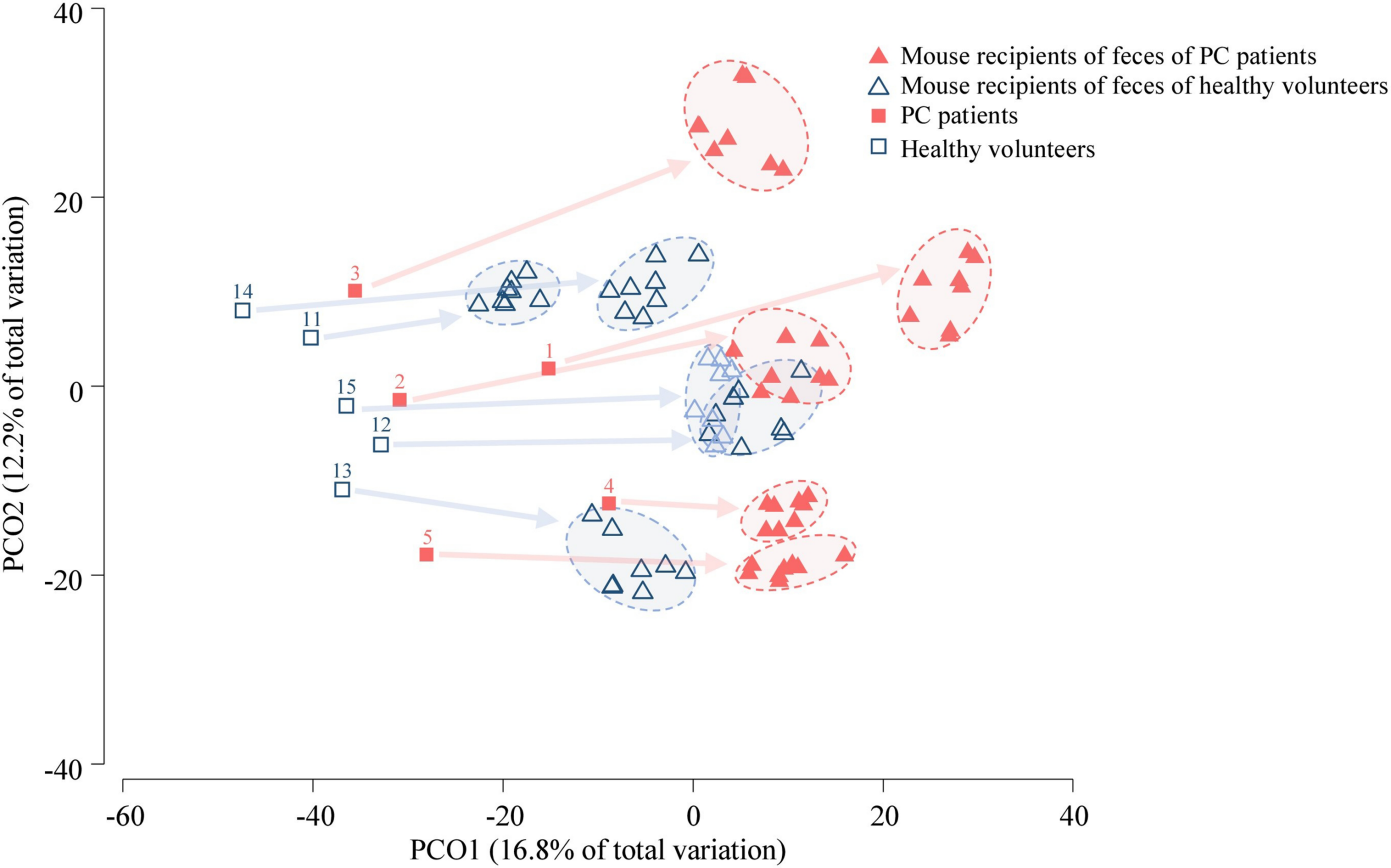
PCoA of human (n = 10) and mouse fecal microbiota (n = 20). The samples from a pair of mice who were transplanted from the same human donor, and collected at four time-points are surrounded by an oval (4 samples per mouse, 8 mouse samples for one human donor). The numbers correspond to human donors. Arrows link the human donor sample to fecal samples from corresponding transplanted mice. The analysis was based on the relative abundance of species.

**Figure 3:**
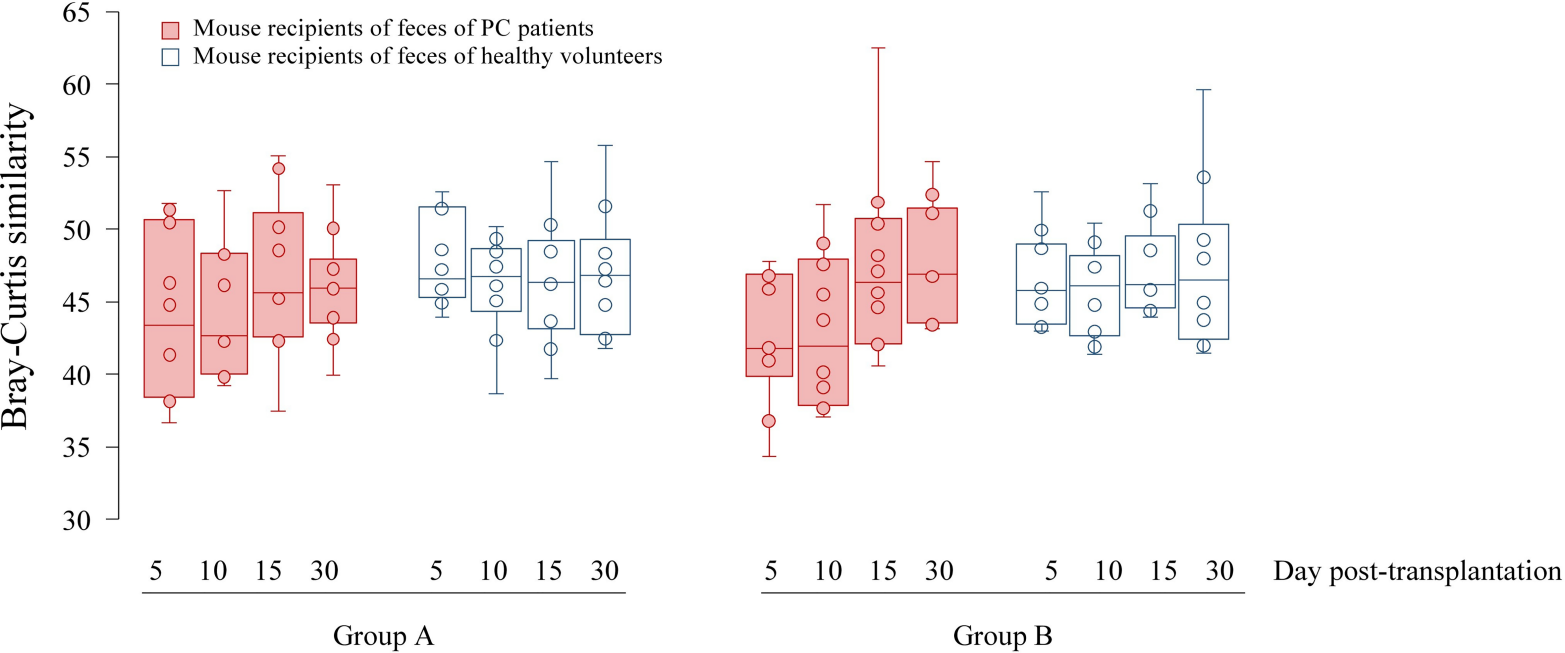
Boxplots showing Bray-Curtis similarities of fecal microbial communities of mice on day 5, 10, 15 and 30 after transplantation. Since each human feces were transplanted into two mice denoted Group (A, B), microbiota analyses were performed twice.

Six genera had a relative abundance > 10% in at least one (of 10) human stool samples. *Bacteroides*, *Blautia* and *Prevotella* surpassed the 10%-threshold in 8, 4 and 2 samples, respectively, while *Alistipes*, *Streptococcus* and *Succinivibrio* each surpassed it in one sample (**Supplemental Table 3**). Principal coordinates analysis (PCoA) of the Bray-Curtis similarity matrix showed a good separation of PC patients’ and volunteers’ microbiota in the first two PCos (**Figure 2**). Samples from PC patients tended to be displaced rightwards relative to those from controls. Intra-group microbiota similarities, based on relative abundance of species, were more pronounced in controls (median 56.76; interquartile range: 48.80–60.57) than in PC patients (median: 47.53; interquartile range: 44.39–49.69), reaching statistical significance (p=0.01 by Wilcoxon rank sum test) (**Figure 3**). Comparison of microbiota of PC patients *vs.* volunteers revealed several differentially abundant taxa (**Supplemental Table 4**). Of note, *Escherichia coli* and *Streptococcus salivarius* were remarkably more abundant (> 147-fold and > 31-fold, respectively, both in median and mean values) in the fecal microbiota of PC patients than in controls. These differences were reflected at the higher levels of the taxonomic hierarchy up to the phylum (*Escherichia*-Enterobacteriaceae-Enterobacteriales-Gammaproteobacteria-Proteobacteria) and class level (*Streptococcus*-Lactobacilli-Lactobacillales-Bacilli).

### Microbiota of the Human Donors *vs.* Microbiota of the Mouse Recipients

In the PCoA plot including the first two PCos **(** **Figure 2** **)**, mice samples from patient recipients were displaced rightwards relative to those from human donors. Nevertheless, the location of mouse samples on the PCoA correlated with the location of corresponding human donors. Overall, mouse microbiota, compared to that of human donors, was significantly depleted in *Lachnospira*, *Roseburia, Clostridium* and *Coprococcus*_g2, while being enriched in *Anaerofilum* (**Supplemental Table 3**). As shown in **Supplemental Figure 2**, 65 to 99% of recipient mice microbiota was of human donor origin. In contrast, the feces of the human donors shared a lower percentage of microbiota with their corresponding mouse recipients. Shannon diversity index was calculated for the human and mice samples. The diversity index was higher in human samples (cancer patients: 3.59–4.02; controls: 3.43–4.30) than in mice sample (recipients of cancer patients: 2.87–3.79; recipients of volunteers: 3.14–3.92).

### Microbiota of Mouse Recipients of PC Patients *vs.* That of Volunteers

Mouse fecal microbiota were clustered according to the cancer/healthy individual status of their respective donors (**Figure 2**). Samples from mouse recipients of PC patients were displaced rightwards relative to those from mouse recipients of volunteers in the PCoA plot.

These changes were associated with an increased or decreased relative abundance of 43 taxa from phylum to species level, and 260 zOTUs (**Supplemental Table 5**). Importantly, all bacterial species found to be differentially abundant between PC patients and healthy controls as well as between corresponding transplanted mice groups had the same direction of change: the proportions of *Clostridium bolteae*, *Clostridium scindens*, *Clostridium*_g24_unclassified and *Phascolarctobacterium faecium* were higher while those of *Alistipes obesi*, Lachnospiraceae PAC000196_s and Coriobacteriaceae_unclassified species were lower in PC patients and in mice transplanted from these patients. Similarly, of 83 zOTUs differentially abundant between the PC and control groups in both humans and mice, 81 had consisted directions of change (increase or decrease). These zOTUs belong to the species cited above and to 12 additional ones (**Supplemental Table 5**).

The similarity of bacterial communities tended to be lower, although not significantly, among mice transplanted from PC donors than among those whose donors were healthy volunteers (**Figure 3**), which was in line with the trend observed for human samples. The similarity between fecal communities of mice transplanted from PC patients tended to increase over time and reach a level observed in the control mice group.

## Discussion

This pilot study showed that: 1) the fecal microbiota of PC patients was different from healthy controls, 2) mouse recipients of feces from PC patients had lower visceral fat than the recipients of volunteer’s feces, but similar immune/inflammatory parameters, and 3) the microbiota of transplanted mice partially reflected the taxonomic composition of bacterial communities of their corresponding human donor.

Some differences of fecal microbiota between patients with PC and healthy controls are in line with previous studies (Ren et al., 2017; Pushalkar et al., 2018; Half et al., 2019). We observed an increased relative abundance of *Streptococcus* (notably due to *S*. *salivarius*) and *Escherichia* (specifically *E. coli*) in PC patients as compared to healthy volunteers. Pushalkar et al. (2018) also reported a higher proportion of *Streptococcus* in the rectal microbiota of PC patients with biliary obstruction and with higher cancer stages (stage 2 *vs.* stage 1). In addition, PC was associated with an increased proportion of *Escherichia*, Enterobacteriaceae and Proteobacteria, as it was the case in our study. However, Ren et al. found a higher fecal abundance of Bacteroidetes and decreased abundance of Firmicutes and Proteobacteria (although two Enterobacteriaceae members, *Klebsiella* and *Enterobacter*, increased) in PC patients (Ren et al., 2017). Both studies included patients naïve of oncologic treatments, as our study. Differences may be related to the characteristics of the patients (for instance non-oncologic treatments, co-morbidities or genetics), to the diet, to the location of the microbiota sample (rectal *vs.* fecal) or to the metataxonomic analyses.

In our study, the visceral fat, but not the muscle mass, was lower in the mice transplanted with the feces of PC patients as compared to those transplanted with the feces of healthy volunteers. A low visceral fat, which precedes muscle loss, was described in patients with hepatocellular carcinoma and mouse models of this type of cancer (Erdem et al., 2019). The mechanism of visceral fat loss in cancer involves lipolysis by stimulation of the hormone-sensitive lipase and adipose tissue triglyceride lipase which is mediated by cytokines and other lipolytic factors as for instance zinc alpha2-glycoprotein (Bing, 2011; Das et al., 2011). No study has been performed on the link between fat wasting and microbiota in cancer. By analogy with muscle wasting, we could hypothesize that microbiota may stimulate fat wasting by mechanisms involving pro-inflammatory cytokines, pathogen-associated molecular patterns, as lipopolysaccharides, or microbial metabolites as short-chain fatty acids or bile acids (Bindels and Delzenne, 2013).

In our study, we found that mice transplanted with the feces of PC patients had higher levels of bacteria belonging to the *Clostridium* genera. High levels of Clostridia have been associated with the leanness phenotype in mice, as reported by Pedersen et al. (Wang and Hooper, 2019; Petersen et al., 2019). They showed that specialized lymphoid T cells (T follicular helper) stimulated the production of IgA by lymphoid B cells which cross the epithelial barrier. In the gut lumen, these IgA could inappropriately target some Clostridia species and promote their proliferation, but antagonize the proliferation of beneficial Clostridia. As a result, the expression of genes involved in lipid synthesis and absorption decreased, which ultimately lead to lower weight and fat mass (Petersen et al., 2019). The authors did not find any impact of T follicular helper cells on cecal short-chain fatty acid, suggesting this effect to occur independently of the short-chain fatty acid production. The impact of other bacteria on body composition has been shown in animal studies. Indeed, certain probiotics decrease the fat mass in mouse models of diet-induced obesity (Everard et al., 2014; Lee et al., 2018). Conversely, some bacteria have been involved in cancer cachexia, which is characterized by muscle wasting with or without fat mass loss. In models of colon carcinoma, *Klebsiella oxytoca* was a major stimulant of cancer cachexia (Potgens et al., 2018), but *Lactobacillus reuteri* was beneficial for its prevention (Varian et al., 2016).

The question often arises in what proportion the gut microbiota of human donors engrafts the murine intestine. In our study, the mouse microbiome, when considering zOTUs with a relative abundance > 0.1%, was in large part (between 65 and 99%) of human origin. The fact that it did not reach 100% could be due to zOTUs which were present in donors below the detection levels and/or to contamination, for instance through food, as the mice were raised in SPF conditions. Other studies evaluating the impact of a single human transplantation described a recovery of 85% of human genus-level taxa in GF mice (Turnbaugh et al., 2009), whereas 59% of the fecal microbiota of antibiotic-treated mice were attributable to the donor bacterial community (Staley et al., 2017), several weeks after transplantation. Riquelme et al. focused on PC and transplanted antibiotic-treated mice with fecal samples from patients with PC and healthy controls. The FMT was performed thrice a week for 2 weeks before an orthotopic pancreatic tumor implantation and 1x/week for 5 weeks thereafter. They found that about 40% of the murine fecal microbiome originated from the human donor microbiome, 5 weeks after tumor implantation (Riquelme et al., 2019). The variable FMT protocols and different mouse models may explain partly the different engraftment. A recent study compared the impact of an FMT once a week, with a single FMT or an FMT twice a week, in conventional mice that had underwent bowel cleansing over a duration of 4 weeks. They suggested that the FMT once a week was the best compromise as it led to the highest engraftment of Bacteroidales and *Faecalibacterium* (Wrzosek et al., 2018). In our study, mice had a higher relative abundance of genera *Lachnospira*, *Roseburia, Clostridium* and *Coprococcus*_g2, and a lower proportion of *Anaerofilum* than humans.

Although we hypothesized that inflammatory and immune parameters would be differentially influenced by the source of the gut microbiota used for the FMT (i.e. of PC patients *vs.* healthy donors), we were not able to show that in the present study. Heterogeneity in the microbiome composition between individuals might contribute to hiding differences between the two groups. In addition, the use of GF mice, in which lack of microbiota results in deficient local and systemic immune systems (Round and Mazmanian, 2009), plays a role. Finally, it has been shown that there is a host-specific immune-microbiome interaction (Chung et al., 2012) that might result in a lower ability of human bacteria to influence mouse immune responses.

The strengths of this study are its translational and innovative design, using the feces of PC patients naïve of oncologic treatments and healthy volunteers. The study is limited by the small number of donors, with, by definition, heterogeneous gut microbiota. Despite this, we could demonstrate differences in the gut microbiota in PC patients and healthy volunteers and their corresponding mouse recipients. We found only significant differences in visceral fat mass between mouse recipients of PC patients *vs.* healthy volunteers. Thus, either the gut microbiota truly does not affect the other measured parameters in this situation, or the feces of the donors and their engraftments in mice were too heterogeneous, or the length of follow-up after FMT was insufficient to induce significant changes. Future studies should observe the gut microbiota in PC patients during the progression of their disease and identify the gut microbiota composition associated with the worst changes in metabolic, intestinal and inflammatory parameters and survival. This gut microbiota composition could then be used for FMT into mice in order to prove causality and potentially pave the way for new interventional studies.

## Conclusion

FMT from PC patients into GF mice decreased visceral fat. The resemblance between the microbiota of transplanted mice and their corresponding human donors was partial, but some differences between PC patients and volunteers were common to humans and mice. Further research is required to confirm that feces contain elements involved in metabolic and immune alterations.

## Data Availability Statement

The datasets presented in this study can be found in online repositories. The names of the repository/repositories and accession number(s) can be found below: ENA, PRJEB43581.

## Ethics Statement

The studies involving human participants were reviewed and approved by Ethical Committee of Geneva. The patients/participants provided their written informed consent to participate in this study. The animal study was reviewed and approved by Swiss Federal and the Geneva Cantonal Authorities for animal experimentation study.

## Author Contributions

Conceptualization, LG and JS. Methodology, LG, FH, AM, MT, and JS. Formal analysis, LG, VL, OS, MS, VD, NG, FH, and JS. Investigation, LG, VL, OS, MS, SD, TK, VD, NG, JM, FH, MT, and JS. Resources, LG, AM, MT, and JS. Data curation, VL, OS, MS, SD, and LG. Writing—original draft preparation, LG. Writing—review and editing, VL, OS, MS, SD, TK, VD, NG, JM, AM, FH, MT, and JS. Visualization, LG. Supervision, LG, MT, and JS. Project administration, LG and MT. Funding acquisition, LG. All authors contributed to the article and approved the submitted version.

## Funding

This research was funded by the « Fondation pour l’innovation sur le cancer et la biologie ».

## Conflict of Interest

The authors declare that the research was conducted in the absence of any commercial or financial relationships that could be construed as a potential conflict of interest.

## Publisher’s Note

All claims expressed in this article are solely those of the authors and do not necessarily represent those of their affiliated organizations, or those of the publisher, the editors and the reviewers. Any product that may be evaluated in this article, or claim that may be made by its manufacturer, is not guaranteed or endorsed by the publisher.
