# Supplementary material for: Metataxonomic and Metabolic Impact of Fecal Microbiota Transplantation From Patients With Pancreatic Cancer Into Germ-Free Mice: A Pilot Study
Source: Front Cell Infect Microbiol. 2021 Oct 19;11:752889. doi: 10.3389/fcimb.2021.752889 (PMC8560705; doi:10.3389/fcimb.2021.752889)
Supplement: Supplementary file 1 [file DataSheet_1.docx]

**Supplemental Figure 1. Evolution of glycemia during the oral glucose tolerance test (A) and the insulin tolerance test (B) for the mouse recipients of feces of pancreatic cancer (PC) patients [mean+SD] or healthy volunteers [mean–SD].**

**A**

**B**

**Supplemental Figure 2. The microbiome shared between human donors and recipient mice.** Filled boxplots correspond to the percentage of the human donor reads assigned to zOTUs shared with the recipient mice, while the empty boxplots correspond to the percentage of the mouse sample reads assigned to zOTUs shared with its human donor. For each human donor or mouse sample, we first selected the zOTUs with the relative abundance > 0.1%. The sum of these zOTUs counts was then normalized to 100%. In the hypothetical situation where all the zOTUs reads of human donors engrafted the mouse intestine, we would find a 100% reads shared microbiome in donors. Similarly, if all zOTUs of mice were present in humans, we would find a 100% shared microbiome in mice.
