# Supplementary material for: Metataxonomic and Metabolic Impact of Fecal Microbiota Transplantation From Patients With Pancreatic Cancer Into Germ-Free Mice: A Pilot Study
Source: Front Cell Infect Microbiol. 2021 Oct 19;11:752889. doi: 10.3389/fcimb.2021.752889 (PMC8560705; doi:10.3389/fcimb.2021.752889)
Supplement: Supplementary file 3 [file Table_1.docx]

**SUPPLEMENTAL MATERIAL**

**Figure S1:** Evolution of glycemia during the oral glucose tolerance test (a) and the insulin tolerance test (b) for the recipients of feces of pancreatic cancer patients as mean+SD (gray line) and volunteers as mean–SD (black line).

**Figure S2:** The filled boxplots correspond to the percentage of the human donor reads assigned to zOTUs shared with the recipient mice, while the empty boxplots correspond to the percentage of the mouse sample reads assigned to zOTUs shared with his human donor. For each human donor or mouse sample, we first selected the zOTUs with the relative abundance > 0.1%. The sum of these zOTUs counts was then normalized to 100%. In the hypothetical situation where all the zOTUs reads of human donors engrafted the mouse intestine, we would find a 100% reads shared microbiome in donors. Similarly, if all zOTUs of mice were present in humans, we would find a 100% shared microbiome in mice.

**Table S1:** Plasma cytokines and chemokines of mouse recipients at sacrifice

**Table S2:** Myeloid, T and T reg cells and number of Peyer’s patches of mouse recipients at sacrifice

**Table S3**: Relative abundance of bacterial genera in human and mouse fecal samples.

**Table S4:** Taxa differentially abundant between cancer patients and control subjects.

**Table S5:** Taxa differentially abundant between mice transplanted from cancer patients and those transplanted from healthy controls
